# Dermatologic Lesions with Eosinophilia in the Head and Neck

**DOI:** 10.1007/s12105-025-01757-3

**Published:** 2025-02-25

**Authors:** David T. Danielson, Ian Lagerstrom, Zachary Wary, Aaron Auerbach, David S. Cassarino

**Affiliations:** 1 Department of Pathology, Walter Reed National Military Medical Center, Bethesda, MD USA; 2 Joint Pathology Center, Silver Spring, MD USA; 3 Department of Pathology, Kaiser Permanente Los Angeles Medical Center, Los Angeles, CA USA

**Keywords:** Eosinophilia, Dermatologic pathology, Head and neck pathology

## Abstract

**Background:**

Dermatologic lesions with notable eosinophilic infiltration of the head and neck region represent a diverse group of conditions, ranging from benign to malignant proliferations.

**Methods:**

We performed a comprehensive literature review focusing on head and neck dermatologic conditions that commonly present with a prominent eosinophilic infiltrate.

**Results:**

This review provides an overview of common entities showing prominent associated eosinophilic inflammatory infiltrates in this region, including epithelioid hemangioma, eosinophilic cellulitis (Wells syndrome), eosinophilic folliculitis, eosinophilic granulomatosis with polyangiitis (Churg-Strauss syndrome), granuloma faciale, and Langerhans cell histiocytosis (LCH).

**Conclusion:**

Eosinophils play a key role in the pathogenesis of these disorders, although the exact mechanisms remain poorly understood. Accurate diagnosis is crucial for differentiating these conditions, as they can share similar histologic features. This review aims to enhance understanding of these eosinophilic dermatologic conditions, improving diagnostic accuracy and treatment strategies.

## Introduction

The head and neck region is frequently affected by dermatologic conditions characterized by eosinophilic infiltration. Each can present with similar histologic features but differ significantly in their clinical behaviors, ranging from benign and self-limiting to severe and life-threatening. The presence of eosinophils in these conditions provides a valuable diagnostic clue, yet challenges remain in distinguishing between the different entities based upon clinical and/or histopathologic findings alone. This review article aims to provide a comprehensive overview of the most common eosinophilic dermatologic lesions in the head and neck area, including epithelioid hemangioma (EH), eosinophilic cellulitis (EC), eosinophilic pustular folliculitis (EPF), eosinophilic granulomatosis with polyangiitis (EGPA), granuloma faciale (GF), and Langerhans cell histiocytosis (LCH). By examining clinical presentations, histopathological features, and potential differential diagnoses, the authors aim to guide clinicians and pathologists in the accurate identification and management of these complex disorders. Furthermore, we explore the underlying immunologic and inflammatory mechanisms that drive eosinophil infiltration in these conditions and discuss emerging therapeutic strategies.

## Epithelioid Hemangioma

EH, also known as histiocytoid hemangioma and angiolymphoid hyperplasia with eosinophilia, typically affects adults in their third to fifth decades of life with no clear sex predominance. Most lesions occur in the head and neck region with roughly one third of cases occurring around the ear [1]. EH presents as one or more red, pink, or brown papules/nodules that can coalesce to form a plaque and are typically less than one centimeter in greatest dimension [2, 3]. EH is often asymptomatic, although pruritus and bleeding may be present [4]. Peripheral eosinophilia is uncommon, but may be present in a small subset of cases [5].

**Fig. 1:**
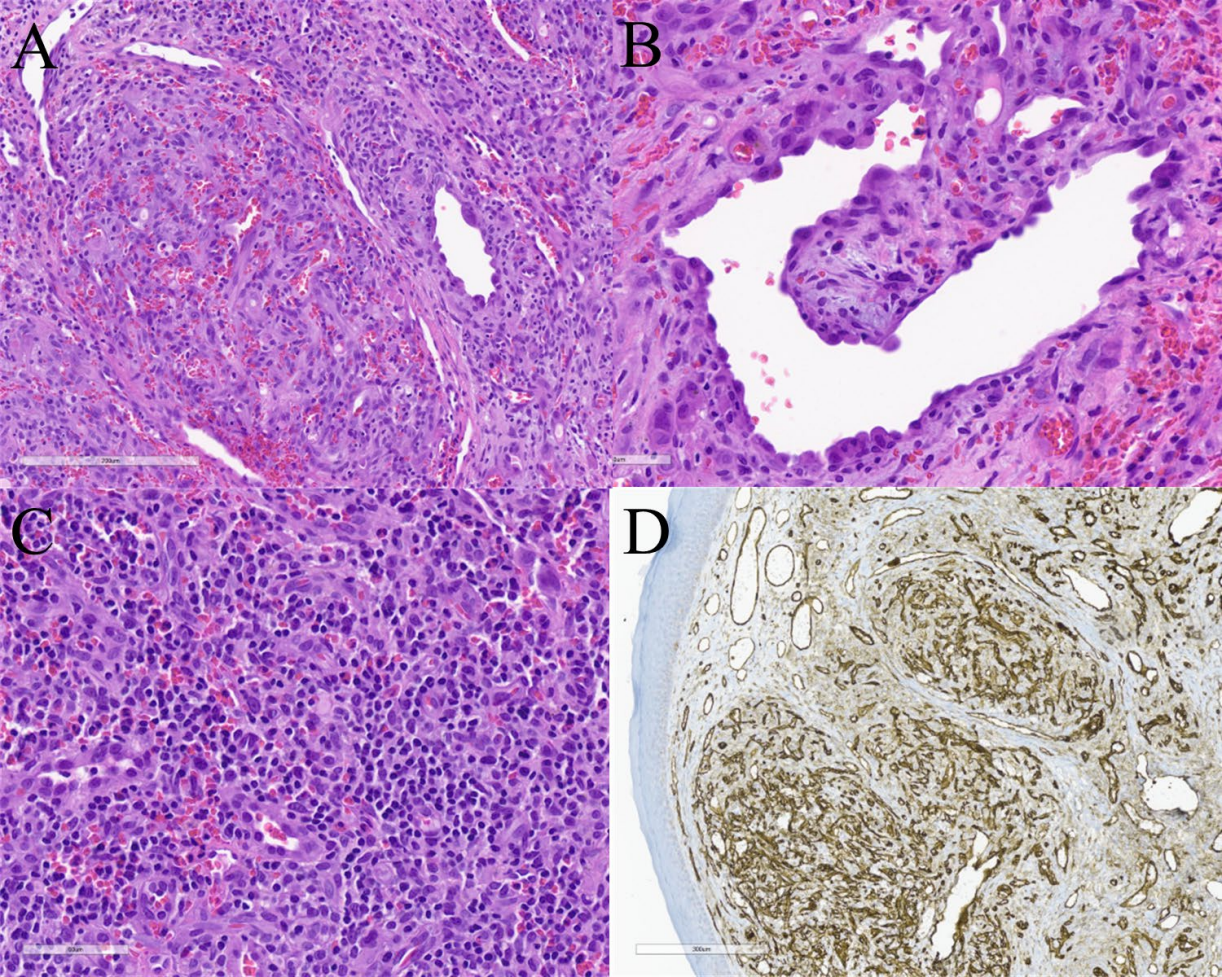
Epithelioid hemangioma. **A** Lobules of variably sized vessels lined by plump endothelial cells which range from slit like to large and dilated with a background predominantly lymphocytic inflammatory infiltrate (original magnification, ×200). **B** Vessel lined by plump endothelial cells that show a “hobnailed” appearance. The endothelial cells lack nuclear atypia and mitotic figures are not appreciated (original magnification, ×400). **C** The background inflammatory infiltrate is composed of predominantly small lymphocytes with scattered eosinophils (original magnification, ×400). **D** CD31 immunohistochemical staining highlights the endothelial cells and emphasizes the lobular architecture (original magnification, ×100)

Microscopically, EH typically appears as a well-circumscribed dermal (or deeper) proliferation of variably sized blood vessels in a distinctive lobular pattern with characteristic plump to epithelioid-appearing endothelial cells that often protrude into the lumen of vessels creating a “hobnailed” appearance (Fig. 1) [2, 3]. The vessels are surrounded by a dense mixed inflammatory infiltrate that is predominantly composed of lymphocytes and eosinophils [2, 3, 6, 7]. The lymphoid infiltrate can vary between cases and some cases will have lymphoid follicles. EH cases also vary over time with the lymphoid infiltrate typically becoming more prominent in the later stages of the disease [3]. Rare mitotic figures may be present in angiogenic foci but atypical mitotic figures should not be seen [2]. Fibrosis may be prominent in older lesions.

The pathogenesis of EH has not been fully elucidated, without consensus in the literature as to whether it is a reactive or neoplastic process. Many cases of EH have been observed to occur in close proximity to a damaged vessels, suggesting a reactive process that arises due to vascular damage and subsequent repair [8]. However, arguing against a reactive process, Wilms tumor 1 (WT1) immunoreactivity, which is seen in many vascular neoplasms, has also been seen in a majority of EH cases [9]. Moreover, endothelial cells of the tumor frequently show nuclear FOS and/or FOSB immunostaining pattern, and a subset of cases have a *FOS* or *FOSB* gene rearrangement detectable by florescence in-situ hybridization (FISH) [10–13], findings suggestive of a neoplastic process. The exact mechanism of FOS and/or FOSB overexpression in EH cases that lack a detectable rearrangement remains unclear, but it may be the result of epigenetic modifications, point mutations, or histone modifications [13].

EH can have histologic overlap with both epithelioid angiosarcoma (EAS) and Kimura disease. EAS has increased mitotic activity and shows a higher degree of cytologic atypia compared to EH. Furthermore, necrosis and areas of anastomosing vessels or a sheet-like, confluent growth pattern are often present in EAS, but should not be present in EH [14]. EAS may be associated with a mild inflammatory infiltrate, but will not typically present with the brisk lymphocytic and eosinophil rich infiltrate seen in EH [15]. Although follicles may be present in EH, Kimura disease often has more prominent follicles with follicular hyperplasia and will demonstrate interfollicular eosinophilic abscesses [16]. Eosinophilic follicle lysis and hyaline material in germinal centers (due to IgE deposition) are often present [17]. Neoangiogenesis may be present in Kimura disease, but it should lack the plump to epithelioid endothelial cells seen in EH [18]. Elevated serum levels of IgE and peripheral blood eosinophilia are present in nearly all cases of Kimura disease, but are uncommon in EH [19].

Surgical resection is the most common treatment for EH, although other treatment options are available and include corticosteroid injections, cryotherapy, pulsed dye laser therapy, and carbon-dioxide laser therapy [1, 20–22]. Regardless of the treatment method, local recurrence is common and occurs in the majority of cases. Of the described methods, local recurrence was lowest in cases treated with surgical excision, with a treatment failure rate of 40.8% and a mean disease-free survival of 4.2 years [1].

## Eosinophilic Cellulitis

EC, also known as Wells syndrome, is a rare inflammatory dermatosis with a strikingly eosinophil-rich inflammatory infiltrate. This dermatosis was first described in 1971 as a recurrent granulomatous dermatitis with eosinophilia and later designated EC [23, 24]. There is no clear sex predominance, and it occurs at a mean age of 33.6. Clinically, it presents with large, erythematous plaques that can be either localized or diffuse. Systemic symptoms, including fever and malaise, may be present in a subset of cases [25]. Peripheral eosinophilia occurs in roughly 67% of cases, and leukocytosis is present in approximately 40% of cases [25, 26].

**Fig. 2:**
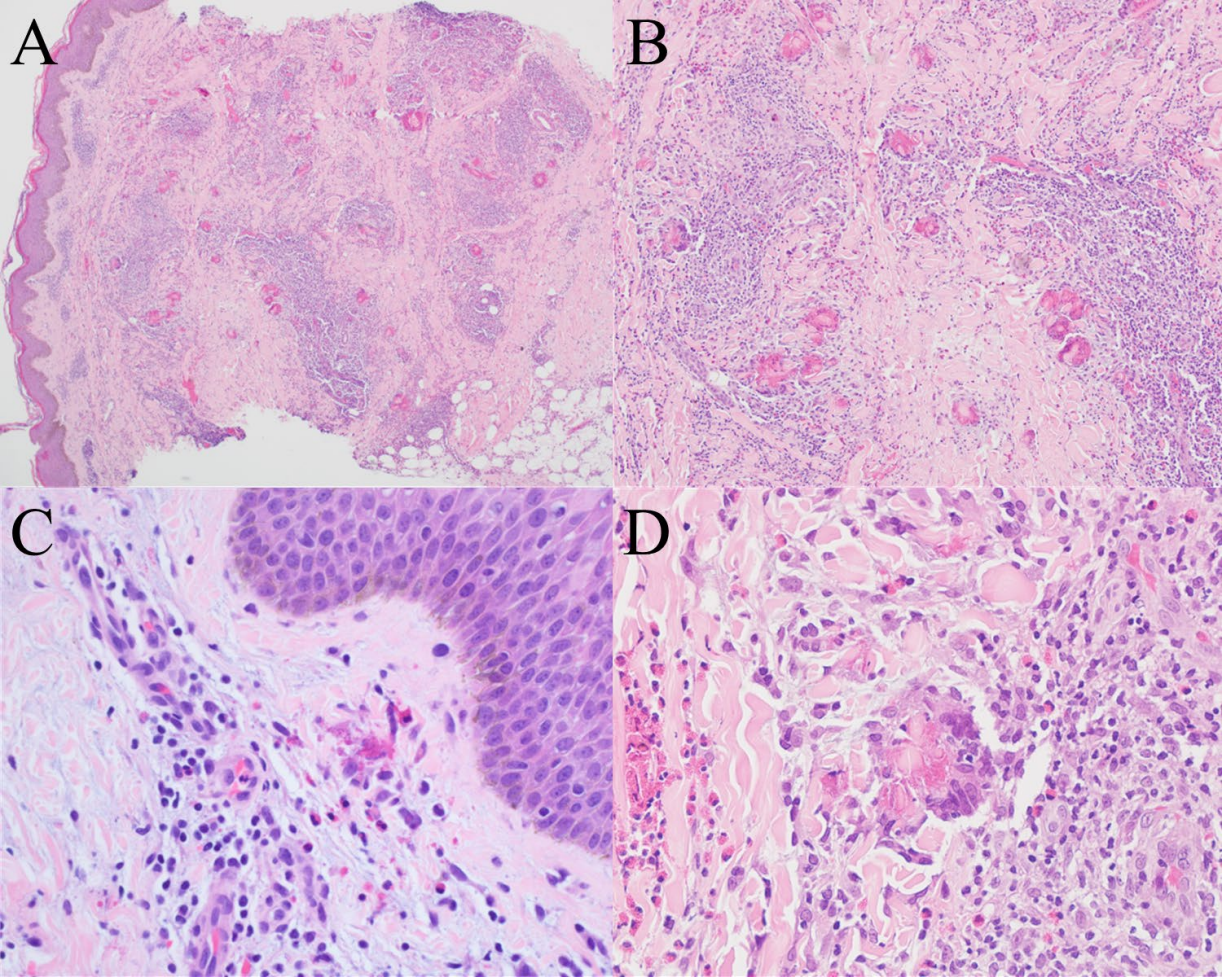
Eosinophilic cellulitis. **A** Low power image demonstrating a superficial and deep perivascular and interstitial inflammatory infiltrate with extension into the subcutis (original magnification, ×40). B, Higher power image showing the inflammatory infiltrate that is made up of numerous eosinophils with admixed lymphocytes and histiocytes (original magnification, ×200). **C** and **D** Flame figures are appreciated in the dermis with surrounding eosinophils (C, original magnification, ×400) and surrounding palisaded histiocytes (**D**, original magnification, ×400)

Histologically, EC presents with dermal edema and a diffuse eosinophilic infiltrate throughout both the superficial and deep dermis and occasionally the subcutis [24, 27, 28]. Epidermal spongiosis may be present in some cases and flame figures are often seen, which are formed by eosinophilic major basic protein from eosinophil granules depositing on collagen fibers (Fig. 2) [27, 28]. Flame figures are a characteristic of EC, but they are not specific as they can be seen in other eosinophilic dermatoses [29]. The histologic appearance of the disease can change over time with later stages of the lesion showing a decreased eosinophilic infiltrate and the appearance of granulomatous inflammation surrounding the flame figures [27, 28].

The pathogenesis of EC is not fully understood. Cases of EC have been reported following triggering events including insect bites, viral infection, vaccination, parasitic infection, and mediation use [30–34]. EC cases have also been reported in association with an underlying disorder including hematologic and nonhematologic malignancies [35, 36], and a subset of cases are idiopathic with no identifiable triggering event or underlying disease [37–39]. The common relationship to a triggering event or underlying conditions hints that EC may be a hypersensitivity mediated reaction. CD4 + T helper (Th) cells play a key role in hypersensitivity reactions and EC has been linked to an increase in IL-5 expressing Th2 cells [40]. Moreover, CD163 + and CD206 + M2 macrophages which promote the Th2 response have been shown to be present in the mixed inflammatory EC infiltrate [41, 42]. Cytokines secreted by Th2 cells, such as IL-5, are crucial for eosinophil development, expansion, and survival [43]. Eosinophils, when recruited, are thought to secrete cytokines IL-4 and IL-13, which promote and enhance the Th2 response [44]. Eosinophils in patients with hypereosinophilic syndromes such as EC have been shown to express the alpha chain of the IL-2 receptor (CD25) and can be “primed” for granule release through binding of IL-2 (produced by activated T-cells) [45].

The differential diagnosis for EC includes other dermatologic conditions that may present with erythematous and indurated skin lesions. Bacterial cellulitis and the early stages of necrotizing fasciitis can have an overlapping clinical appearance with EC; however, the lesions will typically be painful, and the patient will have systemic symptoms such as fever. Necrotizing fasciitis will also show some response to antibiotic therapy or debridement, unlike EC [46]. Histologically, bacterial cellulitis and the early stages of necrotizing fasciitis have prominent dermal neutrophilic infiltrates, as opposed to the characteristic eosinophilic infiltrates, often with flame figures, seen in EC [47]. Another important item to consider in the differential is EGPA. The cutaneous manifestations of EGPA may have a similar appearance to EC; however, EGPA should have additional clinical findings, which can include asthma, paranasal sinusitis, pulmonary infiltrates, mononeuropathy, and/or polyneuropathy [48]. Microscopically, vasculitis should be present in EGPA and absent in EC [34]. EGPA will be discussed further in a later section.

The most common treatment option for EC is oral steroids, although topical steroids may be successful in the treatment of localized lesions [25, 49]. Cyclosporine is an immunosuppressant that suppresses Th cell function, a key contributor to eosinophil recruitment and activation, which has been used in some cases of EC [50, 51]. A variety of other treatment options have been reported in the literature as an alternative to steroids, which include dapsone, tacrolimus (oral or topical), antihistamines (often in combination with other treatment options), interferon alpha, and tumor necrosis factor (TNF)-alpha inhibitors [49]. This treatment list is not exhaustive.

## Eosinophilic Pustular Folliculitis

EPF, previously known as Ofuji disease, is a noninfectious, recurrent dermatosis that was first described in 1970 by Ofuji et al. [52]. EPF has a predilection for males and most commonly occurs in the third and fourth decades of life [53]. There are three well-described variants of EPF depending on the demographic involved: immunosuppression-associated EPF, infancy-associated EPF, and classic EPF [54]. The immunosuppression-associated variant of EPF most commonly occurs in individuals with HIV, but can also occur in other immunosuppressive conditions such as malignancy [27]. Infancy-associated EPF presents at a mean age of 6.1 months of life and retains the male predilection seen in the other variants [55]. Classic EPF presents in otherwise healthy adults without evidence of immunosuppression [56]. Clinically, EPF is characterized by recurrent eruptions of pruritic follicular papules and pustules that can coalesce into plaques and are most commonly localized to the face, although lesions on the truck, hands, and feet also occur [57, 58].

**Fig. 3:**
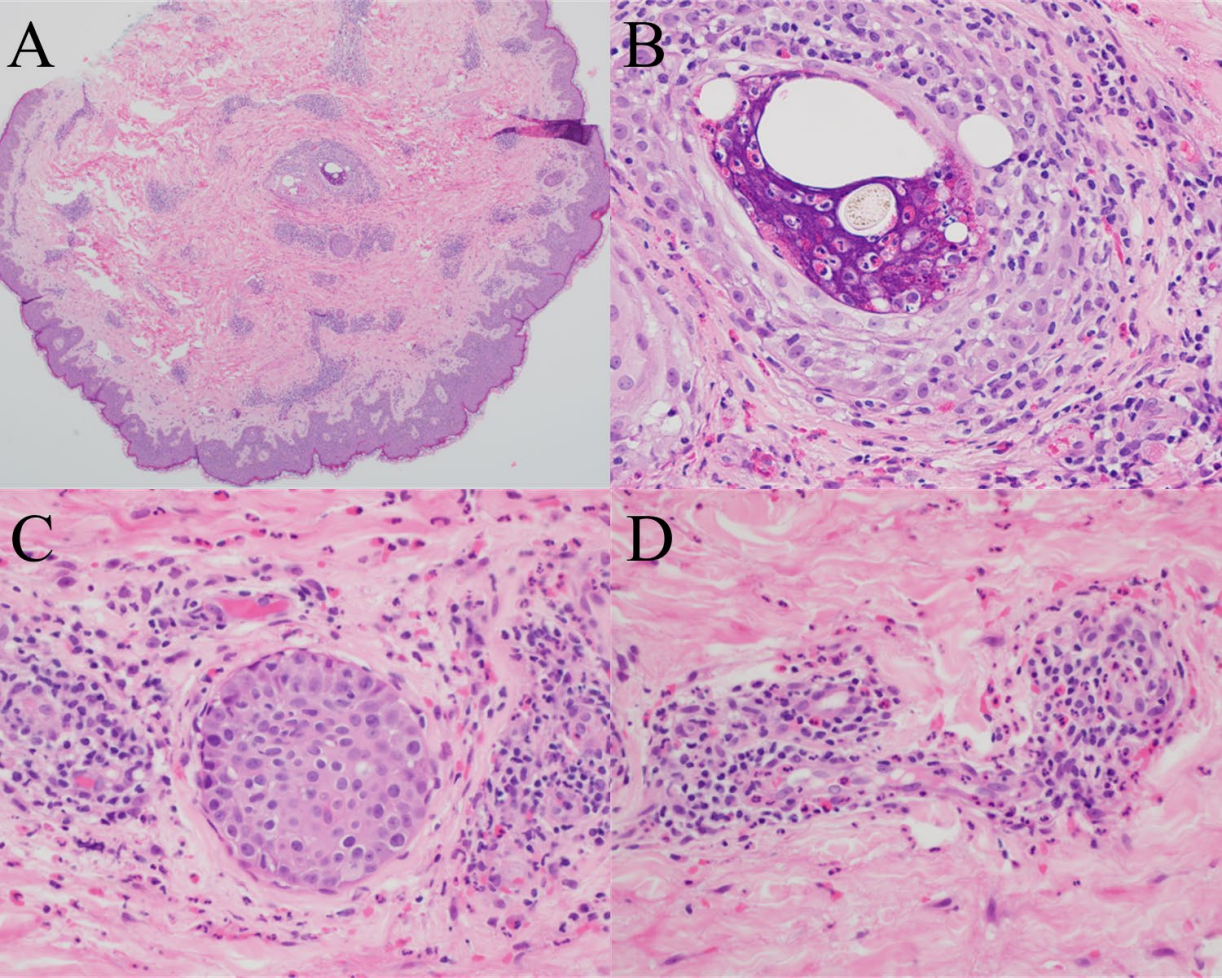
Eosinophilic pustular folliculitis. **A** Unremarkable epidermis with dermal perivascular inflammation and folliculitis with intrafollicular abscess (original magnification, ×40). **B** Hair follicle with significant eosinophilic infiltration and surrounding perifollicular mixed inflammation with eosinophils (original magnification, ×400). **C** Perifollicular mixed inflammation with eosinophils (original magnification, ×400). **D** Perivascular mixed inflammation with eosinophils, which alone is non-specific (original magnification, ×400)

Microscopically, EPF is characterized by an infiltration of many eosinophils into the hair follicles, frequently involving the infundibulum with intraluminal abscess formation (Fig. 3). Eosinophils may also be seen in the associated sebaceous glands and ducts [54]. Spongiosis is typically present and intraepithelial lymphocytes and eosinophils are often found in the surrounding epidermis [27, 54, 57, 58]. Follicular eosinophilic and neutrophilic abscesses may be present in a subset of cases [54, 58]. Cases of immunosuppression-associated EPF will have a nearly identical histologic appearance, with some reports of abundant perifollicular mast cells and a predominant CD8 + T-cell population in the HIV-associated subtype [59, 60]. In infancy-associated EPF, true folliculitis is not always present, and the eosinophilic infiltrate may instead be mostly perifollicular and/or perivascular [61, 62]. A dense interstitial and/or perivascular dermal eosinophilic infiltrate is present in some cases of infancy-associated EPF with associated flame figures present in roughly a quarter of cases [55, 61, 62].

The exact etiopathogenesis of EPF is unknown, although it has been hypothesized that EPF may be a Th2 cytokine dependent condition. This is supported by a case of EPF that demonstrated a positive response to cyclosporin and a subsequent decrease in the mRNA expression levels of Th2 cytokines (IL-4, IL-5, IL-13, IFN-γ, and IL-17) in peripheral blood mononuclear cells (PBMC) following treatment [63]. Similarly, EPF lesions show a positive response to recombinant IFN-γ, and IL-5 levels also decrease in PBMCs following therapy [64]. A Th2 dependent response is further supported by demonstration of an abundant CD163 positive M2 macrophage interstitial and perifollicular infiltrate and the presence of IL-10 and STAT6 positive infiltrating cells in EPF [65]. Interestingly, the infiltrating eosinophils in EPF produce prostaglandin D2 (PGD2), which in turn increases eotaxin-3 mRNA expression in human sebocytes [66]. Eotaxin is an eosinophil chemoattractant, and expression in sebocytes helps explain the predominant follicular and perifollicular eosinophilic infiltrate seen in EPF [66, 67].

The differential diagnosis for EPF can be challenging due to its overlapping clinical and histologic features with various other dermatologic conditions. Clinically, the pruritic papules and pustules in classic EPF may be confused with fungal folliculitis or demodex folliculitis, which both show folliculocentric inflammation, but can usually be differentiated microscopically by the identification of the microorganisms on routine hematoxylin and eosin or fungal stains and the absence of a prominent eosinophilic infiltrate [57, 68]. Additionally, acne, rosacea, seborrheic dermatitis, folliculocentric drug hypersensitivity reactions, and folliculotropic T-cell lymphoproliferative disorders can share similar clinical manifestations to EPF, but these entities also lack the prominent follicular eosinophilic infiltrate of EPF [57, 69].

In infants and children, the differential diagnosis expands to include conditions like erythema toxicum neonatorum and transient neonatal pustular melanosis. These conditions are self-limiting neonatal dermatoses with eosinophilic and/or neutrophilic pustules that can mimic the pustules seen in infancy-associated EPF [54, 55]. Infantile acropustulosis can be clinically and microscopically indistinguishable for infantile-EPF, with some authors suggesting that they may be different presentations of the same disease [55, 70]. LCH, which may present with papules and pustules in young children, is also in the differential diagnosis. LCH can usually be differentiated microscopically and by immunohistochemistry due to the neoplastic histiocytic infiltrate [27, 55].

The first line therapy for EPF is typically systemic nonsteroidal anti-inflammatory drugs (NSAIDs), such as indomethacin, which is effective in greater than 70% of cases [56, 71]. Topical NSAIDs in combination with topical tacrolimus are also effective in most cases [71]. For cases that do not respond to NSAIDs, numerous other therapeutic options have been described, including ultraviolet phototherapy, interferon therapy, cyclosporine, corticosteroids (topical or oral), minocycline, isotretinoin, and dapsone [27, 58, 63, 64, 71].

## Eosinophilic Granulomatosis with Polyangiitis

EGPA is a rare, anti-neutrophil cytoplasmic antibody (ANCA)-associated vasculitis (AAV) affecting small-to-medium sized vessels. Previously known as Churg-Strauss syndrome, the clinical presentation is variable and multiple diagnostic criteria currently exist [48, 72–74]. It is characterized by adult-onset asthma, blood and tissue eosinophilia, and small-to-medium vessel necrotizing vasculitis [75]. Clinical criteria include obstructive airway disease, nasal polyps, and mononeuritis multiplex. Laboratory criteria include blood eosinophilia, extravascular eosinophilic predominant inflammation, p-ANCA or antiproteinase-3 antibodies, and hematuria [74]. Furthermore, EGPA can be subclassified based on the ANCA status (either ANCA-positive or ANCA-negative) [76]. This condition can manifest in the skin in approximately 40% of cases [77]. Skin manifestations include purpura, nodules, urticaria, livedo, and ulcers. EGPA most commonly affect the lower limbs; however, the head and neck can also be involved [78, 79].

The pathogenesis of EGPA is driven by both genetic and environmental factors. Genetically, ANCA-positive disease is associated with HLA-DQ, and ANCA-negative disease is associated with mutations in GPA33 and IL-5 [76]. Environmental factors that increase the risk of developing EGPA include exposure to silica, organic solvents, and farming, while smoking decreases the risk [80]. Eosinophils play an important role in the pathogenesis of EGPA, although the exact mechanisms of eosinophil-mediated inflammation are not fully understood [81].

Recent advancements in the understanding of the pathogenesis of EGPA have led to new treatment options, including therapies that target B-cells, such as rituximab, and eosinophilic cytokines, such as IL-5 [82]. However, glucocorticoids remain an important component of therapy [76]. Treatment of EGPA was historically suggested to be dependent on ANCA status, but newer evidence did not reveal any significant difference in response to therapies between ANCA-positive and ANCA-negative groups [76, 83].

**Fig. 4:**
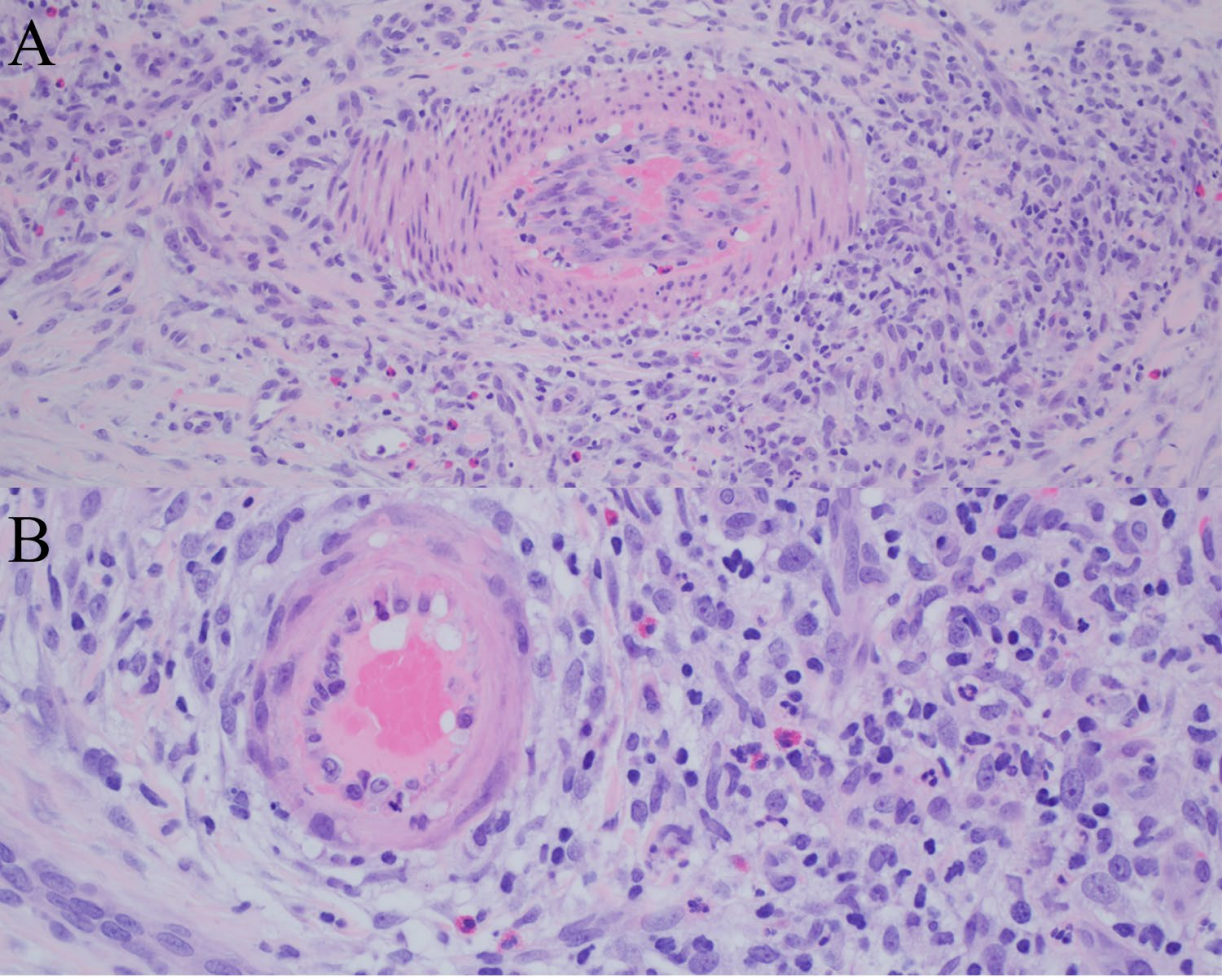
Eosinophilic granulomatosis with polyangiitis. A and B, Prominent perivascular and intravascular inflammation involving the dermal arterioles with inflammatory infiltrate that is composed of eosinophils, neutrophils, lymphocytes and histiocytes. In A, a central fibrin clot is appreciated (original magnification ×200 and ×400, respectively)

Biopsy is the gold standard for assessment of cutaneous vasculitis. Evaluation is based on the vessel size predominantly involved, the extent of involvement, the inflammatory cell type mainly mediating the damage, and incorporation of other relevant studies such as direct immunofluorescence [79]. Skin lesions in EGPA are characterized by necrotizing vasculitis of small to medium-sized vessels associated with granulomas and increased tissue eosinophils (Fig. 4) [78]. The deep dermal and subcutaneous vessels are typically involved, and direct immunofluorescence is often negative. Eosinophilic or ‘red’ extravascular granulomas may be present, which are palisaded neutrophilic granulomas with eosinophils [84]. These findings are not specific to EGPA, and correlation with the other clinical and laboratory findings is necessary to make the diagnosis.

Cutaneous vasculitis has a broad differential diagnosis, which will not be entirely covered in this review. Granulomatosis with polyangiitis and microscopic polyangiitis are the other AAV entities to consider. These diseases affect similarly sized vessels to EGPA; however, they do not typically present with blood and tissue eosinophilia and are not associated with asthma [85]. Drug-related vasculitis is the most common form of vasculitis, and can present with prominent tissue eosinophilia, although it typically manifests as a leukocytoclastic vasculitis [81, 86].

## Granuloma Faciale

GF is a rare chronic inflammatory skin disease that typically presents as a brown–red plaque on the face, although multiple lesions or extra-facial lesions also occur [87]. Currently, the pathogenesis of GF is not well understood. It has been suggested that GF is a type of chronic leukocytoclastic vasculitis due to the presence of fibrinoid necrosis of small vessel walls identified in a small subset of cases [88]. A study utilizing direct immunofluorescence showed heavy deposition of IgG surrounding vessels in the dermis, which supports that the vessel injury seen in GF is due to the classical pathway activation of complement [89]. This would suggest that the neutrophils and eosinophils present within the lesion are responsible for the vessel injury, and vasculitis may not be part of the pathogenesis of this lesion. GF is frequently unresponsive to therapy, but topical glucocorticoids and tacrolimus remain first line choices [90].

**Fig. 5:**
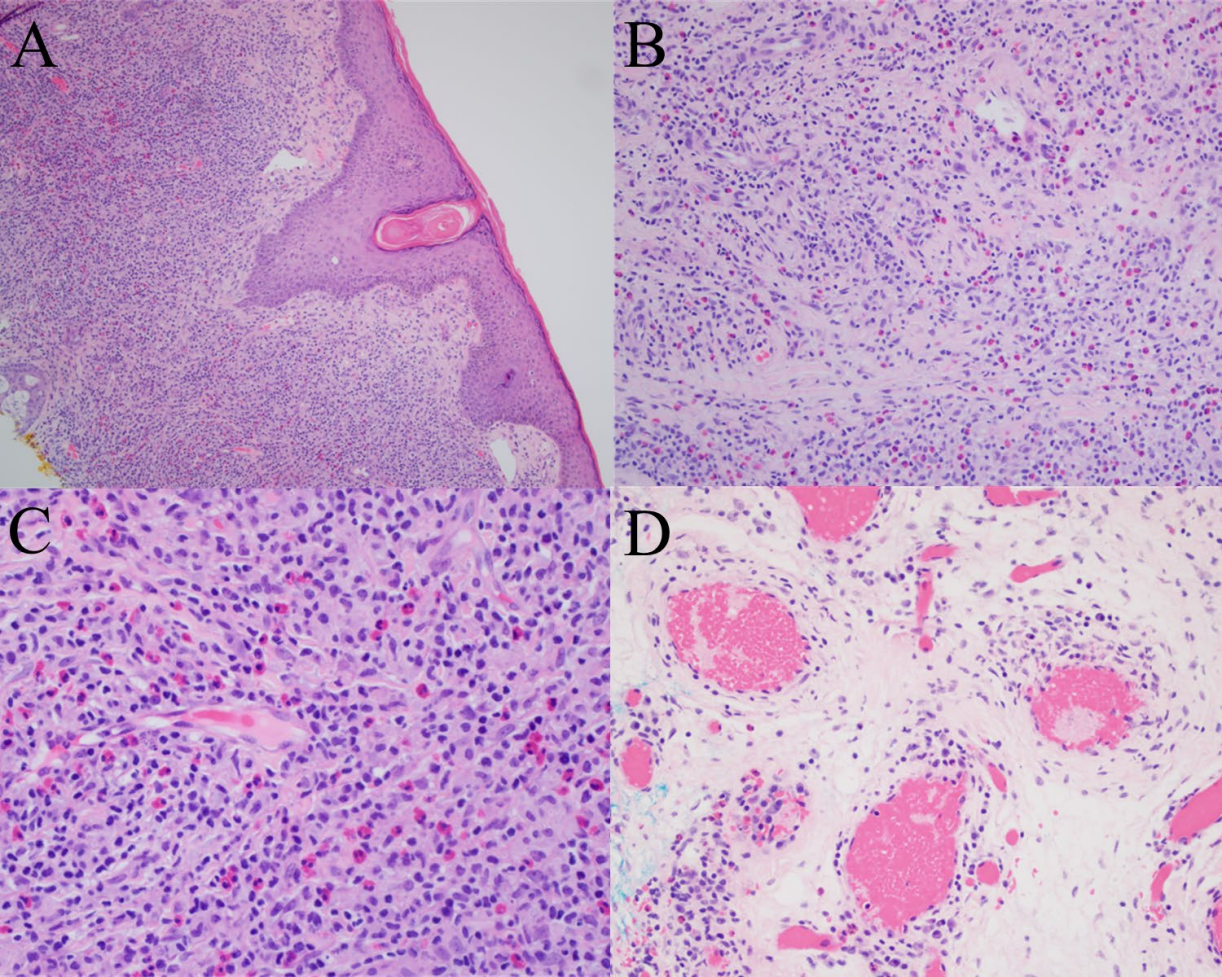
Granuloma Faciale. **A** The papillary dermis under the epidermis has significantly less inflammatory infiltrate compared to the deeper dermis, known as a Grenz zone (original magnification ×100). **B** Foci within the lesion that have a more dense, fibrotic stroma (original magnification ×200). **C** The mixed inflammatory infiltrate is composed of predominantly neutrophils and lymphocytes, with a few eosinophils, plasma cells, and histiocytes in this field. Numerous extravasated red blooe cells and focal leukocytoclastic (karyorrhectic) debris are also present, consistent with an LCV (original magnification ×400). **D** Chronic perivascular changes including organizing fibrosis are present (original magnification ×400)

On histologic examination, GF is characterized by a mixed inflammatory infiltrate composed of many eosinophils, neutrophils, plasma cells, and lymphocytes that is typically separated from the overlying epidermis by a Grenz zone (Fig. 5) [87]. Interestingly, the Grenz zone was historically believed to be a unique feature of GF, but is now known to be a nonspecific finding [91]. As mentioned above, fibrinoid necrosis of vessels and other vascular changes might be seen. Histologic features of GF are on a broad spectrum due to the chronicity of the lesions and can present with both acute and chronic inflammatory patterns with variable amounts of fibrosis [87]. Patterned perivascular fibrosis in particular is often present in chronic lesions and may aid in making the diagnosis.

The differential diagnosis for GF includes other lesions with diffuse mixed inflammatory infiltrates. Erythema elevatum diutinum (EED) is also a chronic leukocytoclastic vasculitis that displays a predominately neutrophilic dermal infiltrate with fibrinoid necrosis of small vessel walls [92]. Similar to GF, EED presents as red-brown plaques, but they are more commonly located on extensor and acral surfaces [92, 93]. These lesions can be histologically indistinguishable from GF, but EED typically shows perivascular fibrosis and has greater numbers of neutrophils and fewer eosinophils and plasma cells [94]. Many consider EED and GF to be the same spectrum of disease, with the latter occurring primarily on the face; although, this assertion is debated in the literature and further studies are needed to clarify the pathogenesis of these conditions. [87, 94].

IgG4-related sclerosing diseases (IgG4-RD) share similar histologic characteristics to GF and EED. Cutaneous lesions in IgG4-RD display small-vessel vasculitis, dermal fibrosis, and a prominent plasma cell infiltrate [95]. An eosinophilic component to the inflammatory infiltrate may also be present. Importantly, the IgG4/IgG ratio in the plasma cell infiltrate needs to be over 40% for the diagnosis of IgG4-RD. It has been suggested that GF and EED might represent localized forms of IgG4-RD due to the amount of histologic overlap [96]. Studies have shown conflicting results following immunohistochemical analysis of the IgG4/IgG ratio of GF cases [95, 96]. Further, cutaneous manifestations of IgG4-RD are rare, and an increased IgG4/IgG ratio alone is not sufficient to make the diagnosis as IgG4-RD as IgG-RD is a clinicopathologic diagnosis that is dependent on a combination of clinical, radiologic, pathologic, and/or laboratory findings [116]. Thus, there is currently not enough evidence to support GF as a cutaneous manifestation of IgG4-RD; however, it is an interesting consideration as further studies are conducted to elucidate the pathogenesis of GF.

Another important differential diagnosis is lymphocytoma cutis or pseudolymphoma, a heterogenous group of T- and B-cell lymphoproliferative processes that can mimic cutaneous lymphoma [97]. Histologically, they are often composed of a dermal infiltrate with many small, mature lymphocytes admixed with other inflammatory cells. A Grenz zone is frequently present, but lymphocyte exocytosis can occur mimicking mycosis fungoides and other T-cell lymphomas [98]. Compared to GF, cutaneous pseudolymphoma lacks vasculitis and usually typically does not have a prominent neutrophilic or eosinophilic infiltrate. Additionally, germinal centers with tingible body macrophages may be present [98].

## Langerhans Cell Histiocytosis

Langerhans cells (LC) are epidermal dendritic cells that play an important role in antigen presentation to T-cells [99, 100]. LCH is a clonal proliferation of cells with LC characteristics and its classification as a neoplasm was cemented after the identification of recurrent BRAF V600E mutations [101]. It is now known that BRAF V600E mutations are the most common and are correlated with high-risk disease and increased resistance to first-line therapy [102, 103]. Other mutations related to activation of the MAPK pathway have been identified, including MAP2K1 (second most common following BRAF V600E), ARAF, BRAF indel, BRAF fusion, and ERBB3 mutations [103]. The etiology remains poorly understood to date and investigations into the cell of origin are ongoing.

LCH is a disease predominately of childhood, but can present at any age [100, 104, 105]. It often presents with a mild clinical course, sometimes resolving spontaneously, but in 20% of disseminated cases it can affect multiple organ systems and be fatal. Cutaneous involvement occurs in approximately 40% of cases and is typically a manifestation of multisystem disease [106, 107]. The skin is the second most commonly involved organ system following bone, but isolated skin involvement is rare [106]. When LCH involves the skin, it manifests as a seborrheic dermatitis-like or eczematous eruption most commonly on the scalp or trunk [106].

For unifocal LCH, observation or local therapies like surgery, intralesional steroids, or radiation are recommended unless it affects specific sites (e.g., nervous system, liver, spleen), where systemic treatment should be used [108]. Multifocal LCH treatments vary, with radiation or bisphosphonates typically used for osseous lesions, topical therapy or methotrexate for cutaneous disease, and chemotherapy for extensive multisystem or brain parenchyma involvement. Refractory cases may need targeted therapies like BRAF/MEK inhibitors [108].

**Fig. 6:**
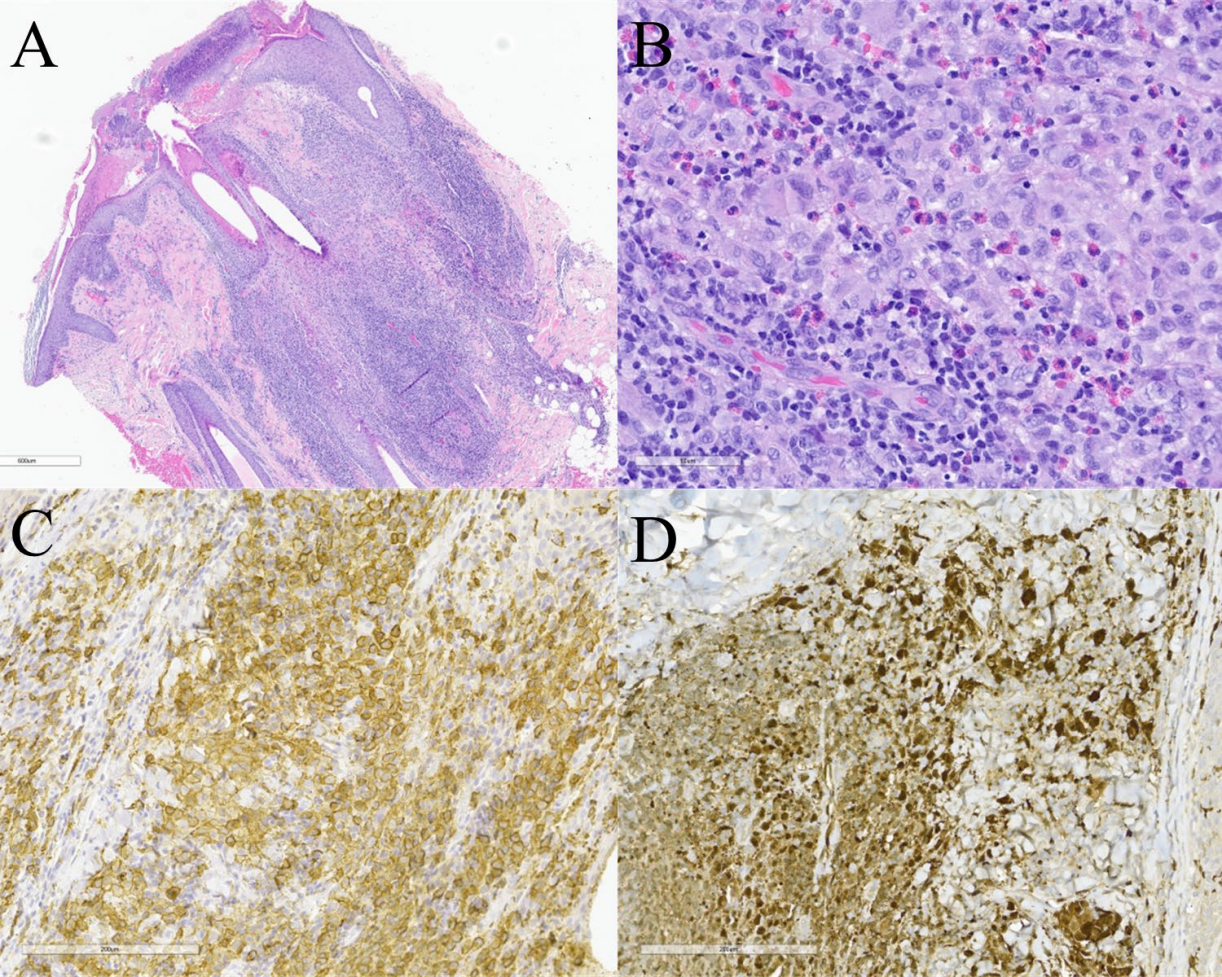
Langerhans cell histiocytosis. **A** The epidermis is excoriated with overlying scale. A vaguely nodular lesion is appreciated underlying the dermis (original magnification ×40). **B** In the dermis, there are numerous medium to large epithelioid cells with abundant pale cytoplasm, irregular to round nuclei, and nuclear grooves. Scattered eosinophils and small lymphocytes are present (original magnification ×400). **C** and **D** The lesional cells stain with immunostains for CD1a and S100, respectively (original magnification ×200)

LCH is characterized by the accumulation of large, round to oval histiocytes with complex nuclear contours with frequent nuclear grooves (Fig. 6). The inflammatory infiltrate typically includes many eosinophils, lymphocytes, and multinucleated histiocytes, with less frequent neutrophils and plasma cells. In active lesions, LCH cells are the predominant cell type, often accompanied by eosinophils. Fibrosis becomes more prominent as lesions progress. By immunohistochemistry, S100, CD1a and CD207 (langerin) positivity is characteristic.

The main consideration in the differential diagnosis of LCH is the accumulation of reactive histocytes occurring in other conditions. Therefore, the differential diagnosis is dependent on the site. In the head and neck, juvenile xanthogranuloma (JXG) is a common non-LCH. It presents as a papule or nodule, often affecting children [109]. Histologically, the lesion is composed of a diffuse infiltrate of histocytes that lack the typical LC characteristics and are negative by CD1a immunohistochemistry. S100 is also notably negative. Eosinophils are usually present scattered throughout the lesion, but are less abundant compared to LCH. Touton giant cells, a type of multinucleated giant cell that have a central eosinophilic core surrounded by a wreath of nuclei, are a notable characteristic of JXG and can be useful in distinguishing it from other cutaneous histiocytoses. The prognosis of JXG is favorable, and it typically regresses spontaneously [110].

Rosai-Dorfman disease (RDD) is another non-LCH that commonly presents as painless bilateral cervical lymphadenopathy that predominately affects children and young adults [111]. Cutaneous RDD is considered a separate disease entity and is more common in older individuals compared to the classic nodal form of RDD [112, 113]. Cutaneous RDD presents as papulonodular lesions, plaques, or eruptive xanthoma-like lesions without a predilection for a specific site [112].

Histologically, cutaneous lesions are remarkably similar to nodal disease and display nodules or sheets of histocytes with abundant eosinophilic cytoplasm associated with lymphocytes, neutrophils and plasma cells. Histocytes with emperipolesis are characteristic and eosinophils are usually absent. CD1a is negative by immunohistochemistry, which is helpful for distinguishing RDD from LCH. JXG is also in the differential diagnosis for cutaneous RDD, but RDD lacks the characteristic Touton giant cells of JXG, and the histiocytes in RDD are positive for S100 and OCT2 [112].

The pathogenesis of RDD is poorly understood, but recently recurrent mutations involving the activation of the MAPK/ERK pathway have been identified in nodal and extranodal RDD, but not in cutaneous RDD [114, 115]. This suggests that RDD is a clonal process, but further studies are needed. The clinical course of RDD is variable, with most of the cases following a benign course. A subset of cases may show a more aggressive clinical course, with involvement of multiple internal organ systems, and require systemic therapy [111].

## Conclusions

In summary, eosinophilic lesions in the head and neck region encompass a wide range of conditions, from benign entities like EPF to neoplasms such as LCH. Tissue eosinophilia represents a valuable diagnostic clue for differentiating these conditions from other conditions of the head and neck skin. Continued research into the mechanisms underlying these conditions and the drivers of eosinophil proliferation and infiltration promises to enhance our ability to further refine diagnostic and therapeutic strategies, potentially benefiting patient outcomes in eosinophilic disorders of the head and neck.

## Data Availability

No datasets were generated or analysed during the current study.

## Declarations

### Disclaimer

The views expressed herein are those of the authors and do not necessarily reflect the official policy or position of Walter Reed National Military Medical Center, Fort Belvoir Community Hospital, the U.S. Army Medical Department, the U.S. Army Office of the Surgeon General, the Department of the Air Force, the Department of the Army, Department of Defense, the Uniformed Services University of the Health Sciences, or any other agency of the U.S. Government. The identification of specific products or scientific instrumentation is considered an integral part of the scientific endeavor and does not constitute endorsement or implied endorsement on the part of the authors, DoD, or any component agency.
